# Characterization of laser speckle flowgraphy pulse waveform parameters for the evaluation of the optic nerve head and retinal circulation

**DOI:** 10.1038/s41598-021-86280-5

**Published:** 2021-03-25

**Authors:** Nobuko Enomoto, Ayako Anraku, Goji Tomita, Aiko Iwase, Takashi Sato, Nobuyuki Shoji, Tomoaki Shiba, Toru Nakazawa, Kazuhisa Sugiyama, Koji Nitta, Makoto Araie

**Affiliations:** 1 Department of Ophthalmology, Toho University Ohashi Medical Center, 2-22-36, Ohashi Meguro-ku, Tokyo, 153-8515 Japan; 2 Tajimi Iwase Eye Clinic, Tajimi, Gifu Japan; 3 Department of Ophthalmology, Kitasato University School of Medicine, Sagamihara, Kanagawa Japan; 4 Department of Ophthalmology, School of Medicine, Toho University, Tokyo, Japan; 5 Department of Ophthalmology, Tohoku University Graduate School of Medicine, Sendai, Japan; 6 Department of Ophthalmology, Kanazawa University Graduate School of Medical Science, Kanazawa, Japan; 7 Fukui-ken Saiseikai Hospital, Fukui, Japan; 8 Kanto Central Hospital of the Mutual Aid Association of Public School Teachers, Tokyo, Japan

**Keywords:** Medical research, Blood flow

## Abstract

To characterize laser speckle flowgraphy (LSFG) pulse waveform parameters for ocular circulation evaluation, a multicenter, prospective, cross-sectional study was conducted in 111 eyes of 86 healthy Japanese individuals. Optic nerve head (ONH) tissue-area, vessel-area mean blur rate (MT and MV, respectively), and MT and MV pulse waveform parameters were obtained using LSFG and ONH structural parameters using planimetry. Multivariate linear mixed-effects modeled regression analysis identified factors contributing to MT- or MV-waveforms using age, gender, smoking history, body mass index, systolic and diastolic blood pressure, heart rate, intraocular pressure, axial length, disc, rim, and β-peripapillary atrophy areas, MT or MV, central retinal artery, and vein equivalents (CRAE and CRVE) as explanatory variables. MT- and MV-waveforms significantly correlated with one or more systemic factors, consistent with previous studies. Following confounding factor adjustment, MT-Skew significantly negatively correlated with β-PPA area (*P* = 0.026); MT- and MV-flow acceleration index positively correlated with CRAE, MT, and MV (*P* = 0.041–< 0.001), compatible with these parameters’ observed correlations to systemic factors. Significantly negative correlations of the blowout score and acceleration time index to CRAE partly conflicted with their correlations to systemic factors, and other waveform parameters showed little correlation to ocular factors. Thus, Skew and flow acceleration index assisted the in vivo ocular circulation characterization.

## Introduction

Disturbed circulation in the optic nerve head (ONH) is related to the development and/or progression of glaucoma^1–3^, and many investigators have studied blood flow impairment in the ONH in glaucoma using various methods, such as scanning laser Doppler flowmetry, color Doppler imaging, or laser speckle flowgraphy (LSFG)^4^. LSFG utilizes the laser speckle phenomenon for the measurement of ocular blood flow in a noninvasive manner^5^ and provides the mean blur rate (MBR), which is a quantitative index of blood flow velocity in the target tissues. For assessing the ONH circulation, the ONH tissue-area MBR (MT) corresponding to the ONH micro-circulation, the ONH vessel-area MBR (MV) corresponding to blood velocity through large visible vessels in the ONH area, and all-area MBR (MA), which is mean MBR in all area of the ONH, can be derived using LSFG^6–9^. Animal experiments using the microsphere^10^ or hydrogen gas clearance method^11,12^ have shown that MT is a quantitative index of the ONH tissue blood flow, and a comparison of the measurement results from LSFG to those from laser Doppler velocimetry has shown that MV indicates blood flow through the major retinal vessels^13–15^. Furthermore, LSFG enables the recording of changes in the pulse waveforms of the measured MBR, such as MT and MV, which are synchronized with the cardiac cycle^6,7,16–21^. Because laser speckle phenomenon is an interference event observed when lasers are scattered by a diffusing surface^22^, the LSFG-measured MBR should also be influenced by reflection, absorption, and penetration depth of the laser in the target tissue^5,13^, and needs to be adjusted not only for systemic factors but also for local ocular factors such as disc, cup, β-PPA area or axial length^23^ for inter-individual comparison. Conversely, LSFG pulse waveforms themselves are relatively free from such effects; therefore, they could be more suited for inter-individual comparison of the ONH or retinal circulatory status than the MT or MV. Several LSFG pulse waveform parameters reportedly showed significant differences between normal and glaucoma eyes^24–26^, between various disease stages of glaucoma^24^, and a significant change after trabeculectomy^27,28^, and water drinking^29^. Further, a significant correlation of leukocyte glutathione levels, an indicator of mitochondrial dysfunction, was found with one LSFG pulse waveform parameter for MT in glaucoma patients^30^. These previous studies have suggested the potential of LSFG pulse waveform parameters in investigating the circulatory status changes in the ONH in relation to glaucomatous damages. In an attempt to characterize the pathophysiological implications of the LSFG pulse waveform parameters, previous studies have correlated them to the circulatory parameters of the systemic circulation. For example, blow-out time (BOT) was reportedly related to the stiffness of large arteries^18–21^, left ventricular function^19^, and systemic vascular resistance^31^; blow-out score (BOS) to the stiffness of large arteries^20^ and left ventricular function^32^; Skew to the stiffness of large arteries^21^; and acceleration time index (ATI) to left ventricular function^32^.

While several studies have looked at the differences of LSFG pulse waveform parameters between the glaucoma and normal eyes^24–26^ or between sexes^6,7,17^, it is still largely unclear which LSFG pulse waveform parameters reflect the pathophysiology of ocular fundus circulation more effectively. We assumed that there should be particularly useful waveform parameters for ocular circulation studies. The objective of this study is to investigate the ophthalmologic implications of seven MT- and MV-LSFG pulse waveform parameters through careful examination of healthy Japanese according to the predetermined uniform measurement protocol and investigate how the known quantitative indices of ocular fundus circulation, that is, MT, MV, and central retinal artery and vein equivalents (CRAE and CRVE) were correlated with seven pulse waveform parameters after adjustment for systemic parameters^6,16,17^ and the ONH structural parameters.

## Results

A total of 111 eyes from 86 healthy participants (57 men and 54 women) with an average age of 47.1 ± 12.2 years were included in the study. Tables 1 and 2 show the baseline characteristics and LSFG parameters of the study participants, respectively. In a preliminary study including 40 normal eyes of a separate group, seven MT- and MV-waveform parameters were twice obtained as described above at an interval of 7 days (by T.S.) and intra-class correlation coefficients between them were calculated. The intra-class correlation coefficients were between 0.967 (95% confidence interval: 0.938–0.982) and 0.999 (95% confidence interval: 0.998–1.000) for MT-waveform parameters and between 0.963 (95% confidence interval: 0.931–0.980) and 0.995 (95% confidence interval: 0.990–0.997) for MV-waveform parameters. Among the MT- and MV-waveform parameters, very high inter-correlations were observed between MT- or MV-BOS and MT- or MV-resistivity index (Spearman’s correlation coefficient: − 0.997– − 0.991) and between MT- or MV-BOS and MT- or MV-Fluctuation (Spearman’s correlation coefficient: − 0.981– − 0.967), indicating that these two parameters were represented by MT- or MV-BOS (Table 3). Table 4 shows the results of the multivariate linear mixed-effect modeled regression analysis assessing the contribution of each factor to the pulse waveforms for MT and MV.

**Table 1:** Characteristics of participants.

Parameters	111 eyes, 86 participants	Range
Age, years	47.1 ± 12.2	30–75
Gender: Men/Woman, no. (%)	57/54 (51.4 / 48.6)	
Smoking history: Yes/No, no. (%)	17/94 (15.3 / 87.4)	
BMI, kg /m	22.1 ± 2.8	17.8–29.1
Systolic blood pressure, mmHg	116.7 ± 14.1	83–148
Diastolic blood pressure, mmHg	72.7 ± 9.4	51–89
Pulse pressure, mmHg	44.0 ± 10.2	24–78
Heart rate, bpm	71.6 ± 8.6	54–96
Spherical equivalent refractive error, diopters	− 1.32 ± 1.97	− 5.8–2.0
Axial length, mm	24.1 ± 1.0	22.2–26.4
Central corneal thickness, µm	529.5 ± 37.2	443–617
Intraocular pressure, mmHg	14.5 ± 2.1	8.0–20.0
Disc area, mm	2.35 ± 0.39	1.50–3.37
Rim area, mm	1.56 ± 0.42	0.75–2.98
β-PPA area, mm	0.40 ± 0.46	0.00–2.19
CRAE, µm	141.5 ± 17.6	106–191
CRVE, µm	222.8 ± 24.8	176–276

The values are represented as mean ± standard deviation. *AU* arbitrary unit, *CRAE* central retinal artery equivalent, *CRVE* central retinal vein equivalent, *MBR* mean blur rate, *MT* MBR in the optic nerve head tissue area, *MV* MBR in the optic nerve head vessel area, *PPA* peripapillary atrophy.

**Table 2:** LSFG parameters of 111 eyes.

LSFG parameter (AU)	MT (range)	MV (range)
MBR	12.6 ± 2.3 (7.4–20.4)	48.8 ± 7.4 (33.9–71.6)
Skew	12.0 ± 1.7 (6.3–15.8)	10.7 ± 1.7 (5.1–14.4)
BOS	76.9 ± 4.8 (62.4–85.1)	80.0 ± 4.4 (65.6–89.1)
BOT	50.6 ± 4.2 (39.4–59.4)	55.0 ± 4.3 (45.2–67.7)
RR	12.7 ± 1.0 (9.9–15.3)	13.1 ± 1.2 (9.8–16.1)
FR	13.1 ± 0.9 (11.2–15.6)	12.4 ± 0.8 (10.7–14.5)
FAI	1.5 ± 0.4 (0.8–3.2)	5.5 ± 1.6 (1.9–10.0)
ATI	29.5 ± 3.3 (21.0–40.5)	29.4 ± 4.7 (15.6–40.8)

The values are represented as mean ± standard deviation.

*ATI* acceleration time index, *AU* arbitrary unit, *BOS* blowout score, *BOT* blowout time, *FAI* flow acceleration index, *FR* falling rate, *MBR* mean blur rate, *MT* MBR in tissue area, *MV* MBR in vessel area, *RR* rising rate.

**Table 3:** Spearman’s rank correlation coefficient between MT- and MV-waveform parameters.

Parameter (AU)		BOS	BOT	RR	FR	FAI	ATI	RI	Fluctuation (%)
Skew	MT	− 0.700*	− 0.776*	0.153	0.735*	0.429*	− 0.329*	0.706*	0.708*
	MV	− 0.569*	− 0.745*	0.042	0.630*	0.391*	− 0.399*	0.588*	0.617*
BOS	MT	–	0.696*	− 0.164	− 0.718*	− 0.550*	0.058	− 0.997*	− 0.981*
	MV	–	0.489*	− 0.136	− 0.479*	− 0.557*	0.172	− 0.991*	− 0.967*
BOT	MT	–	–	0.089	− 0.883*	− 0.185	− 0.042	− 0.697*	− 0.658*
	MV	–	–	0.178	− 0.805*	− 0.056	0.028	− 0.503*	− 0.487*
RR	MT	–	–	–	0.031	0.244*	0.112	0.177	0.205^†^
	MV	–	–	–	0.009	0.163	0.305*	0.149	0.197^†^
FR	MT	–	–	–	–	0.221^†^	0.147	0.711*	0.653*
	MV	–	–	–	–	0.047	0.120	0.472*	0.418*
FAI	MT	–	–	–	–	–	− 0.303*	0.560*	0.597*
	MV	–	–	–	–	–	− 0.488*	0.554*	0.595*
ATI	MT	–	–	–	–	–	–	− 0.069	− 0.085
	MV	–	–	–	–	–	–	− 0.179	− 0.175
RI	MT	–	–	–	–	–	–	–	0.986*
	MV	–	–	–	–	–	–	–	0.971*

*ATI* acceleration time index, *AU* arbitrary unit, *BOS* blowout score, *BOT* blowout time, *FAI* flow acceleration index, *FR* falling rate, *RI* resistivity index, *RR* rising rate.

*P < 0.01, ^†^*P* < 0.05.

**Table 4:** Results of the multivariate linear mixed effect model analysis evaluating the contributing factors to pulse waveforms of MT and MV.

Parameters	Explanatory variable	SBP included^a^		DBP included^b^		SBP and DBP not included^c^	
		Coefficient (*P-*value)		Coefficient (*P*-value)		Coefficient (*P-*value)	
		MT-Skew	MV-Skew	MT-Skew	MV-Skew	MT-Skew	MV-Skew
Skew	Age, years	NS	NS	0.062 ± 0.012 (< 0.001)	0.038 ± 0.013 (0.005)		
	DBP, mmHg			− 0.040 ± 0.017 (0.022)	− 0.047 ± 0.018 (0.011)		
	HR, bpm	NS	NS	− 0.061 ± 0.019 (0.001)	− 0.044 ± 0.020 (0.031)		
	β-PPA area, mm^2^	NS	NS	− 0.617 ± 0.273 (0.026)	NS		

Parameters	Explanatory variable	SBP included^a^		DBP included^b^		SBP and DBP not included^c^	
		Coefficient (*P-*value)		Coefficient (*P*-value)		Coefficient (*P*-value)	
		MT-BOS	MV-BOS	MT-BOS	MV-BOS	MT-BOS	MV-BOS
BOS	Age, years	− 0.149 ± 0.036 (< 0.001)	− 0.079 ± 0.037 (0.037)	− 0.173 ± 0.035 (< 0.001)	− 0.099 ± 0.036 (0.007)		
	DBP, mmHg			0.119 ± 0.049 (0.017)	0.150 ± 0.050 (0.003)		
	HR, bpm	0.186 ± 0.051 (< 0.001)	0.161 ± 0.052 (0.003)	0.139 ± 0.051 (0.008)	0.111 ± 0.052 (0.038)		
	CRAE, µm	− 0.042 ± 0.017 (0.015)	− 0.047 ± 0.018 (0.012)	− 0.038 ± 0.017 (0.026)	− 0.040 ± 0.018 (0.029)		

Parameters	Explanatory variable	SBP included^a^		DBP included^b^		SBP and DBP not included^c^	
		Coefficient (*P-*value)		Coefficient (*P-*value)		Coefficient (*P-*value)	
		MT-BOT	MV-BOT	MT-BOT	MV-BOT	MT-BOT	MV-BOT
BOT	Age, years					− 0.193 ± 0.033 (< 0.001)	− 0.144 ± 0.032 (< 0.001)
	HR, bpm					0.122 ± 0.043 (0.006)	NS

Parameters	Explanatory variable	SBP included^a^		DBP included^b^		SBP and DBP not included^c^	
		Coefficient (*P-*value)		Coefficient (*P-*value)		Coefficient (*P-*value)	
		MT-RR	MV-RR	MT-RR	MV-RR	MT-RR	MV-RR
RR	Gender (Men: 1/ Woman: 0)	NS	NS	NS	− 0.552 ± 0.231 (0.019)		
	Smoking history (Yes: 1/No: 0)	NS	NS	NS	− 0.905 ± 0.014 (0.006)		
	HR, bpm	− 0.040 ± 0.011 (< 0.001)	NS	− 0.041 ± 0.011 (< 0.001)	− 0.048 ± 0.014 (0.001)		

Parameters	Explanatory variable	SBP included^a^		DBP included^b^		SBP and DBP not included^c^	
		Coefficient (*P-*value)		Coefficient (*P-*value)		Coefficient (*P-*value)	
		MT-FR	MV-FR	MT-FR	MV-FR	MT-FR	MV-FR
FR	Age, years					0.035 ± 0.006 (< 0.001)	0.026 ± 0.006 (< 0.001)
	HR, bpm					− 0.026 ± 0.009 (0.005)	NS

Parameters	Explanatory variable	SBP included^a^		DBP included^b^		SBP and DBP not included^c^	
		Coefficient (*P-*value)		Coefficient (*P-*value)		Coefficient (*P-*value)	
		MT-FAI	MV-FAI	MT-FAI	MV-FAI	MT-FAI	MV-FAI
FAI	Age, years	NS	− 0.029 ± 0.013 (0.023)	NS	NS		
	SBP, mmHg	− 0.007 ± 0.002 (0.001)	− 0.032 ± 0.010 (0.002)				
	DBP, mmHg			− 0.014 ± 0.004 (< 0.001)	− 0.067 ± 0.016 (< 0.001)		
	HR, bpm	NS	− 0.036 ± 0.017 (0.041)	NS	NS		
	MT, AU	0.111 ± 0.012 (< 0.001)		0.111 ± 0.012 (< 0.001)			
	MV, AU		0.067 ± 0.017 (< 0.001)		0.074 ± 0.016 (< 0.001)		
	Disc area, mm^2^	− 0.168 ± 0.083 (0.046)		− 0.175 ± 0.081 (0.034)			
	CRAE, µm	0.004 ± 0.001 (0.007)	0.015 ± 0.007 (0.041)	0.004 ± 0.001 (0.015)	NS		

Parameters	Explanatory variable	SBP included^a^		DBP included^b^		SBP and DBP not included^c^	
		Coefficient (*P-*value)		Coefficient (*P-*value)		Coefficient (*P-*value)	
		MT-ATI	MV-ATI	MT-ATI	MV-ATI	MT-ATI	MV-ATI
ATI	Age, years	NS	NS	0.051 ± 0.025 (0.046)	NS		
	Gender (Men: 1/ Woman: 0)	NS	NS	− 2.244 ± 0.612 (< 0.001)	− 3.738 ± 0.902 (< 0.001)		
	DBP, mmHg			0.099 ± 0.034 (0.005)	0.131 ± 0.047 (0.006)		
	CRAE, µm	NS	NS	− 0.031 ± 0.014 (0.027)	NS		

The values indicate estimated partial correlation coefficients ± standard error and those in parentheses indicate *P* values.

*ATI* acceleration time index, *AU* arbitrary unit, *BOS* blowout score, *BOT* blowout time, *CRAE* central retinal artery equivalent, *DBP* diastolic blood pressure, *FAI* flow acceleration index, *FR* falling rate, *HR* heart rate, *MT* mean blur rate (MBR) in tissue area, *MV* MBR in vessel area, *PPA* peripapillary atrophy, *RR* rising rate, *SBP* systolic blood pressure, *NS* not significant.

^a^Only SBP was adopted as an explanatory variable since univariate analysis yielded a *P* value less than 0.2 for both SBP and DBP.

^b^Only DBP was adopted as an explanatory variable since univariate analysis yielded a *P* value less than 0.2 for both SBP and DBP.

^c^SBP and DBP were not adopted as explanatory variables since univariate analysis yielded *P* values > 0.2 for both SBP and DBP.

MT-Skew significantly negatively correlated with diastolic blood pressure (DBP), heart rate (HR), and β-PPA area, and positively with age, while MV-Skew significantly negatively correlated with DBP and HR, and positively with age when DBP was adopted as an explanatory variable.

Both MT- and MV-BOS were significantly negatively correlated with age and CRAE and positively correlated with HR, when the systolic blood pressure (SBP) was adopted as an explanatory variable, and significantly negatively correlated with age and CRAE and positively correlated with DBP and HR when DBP was adopted as an explanatory variable.

MT-BOT significantly negatively correlated with age and positively with HR and MV-BOT negatively correlated with age.

MT-rising rate (RR) was significantly negatively correlated with HR when SBP or DBP was adopted as an explanatory variable, and MV-RR was significantly negatively correlated with gender (greater in women), smoking history (greater with no smoking history), and HR, when DBP was adopted as an explanatory variable. MT- falling rate (FR) significantly negatively correlated with HR and positively with age, and MV- FR was positively correlated with age.

MT-flow acceleration index (FAI) was significantly negatively correlated with SBP and disc area and positively correlated with MT and CRAE, when SBP was adopted as an explanatory variable, and was significantly negatively correlated with DBP and disc area and positively with MT and CRAE, when DBP was adopted as an explanatory variable. MV-FAI was significantly negatively correlated with age, SBP, and HR and positively correlated with MV and CRAE, when SBP was adopted as an explanatory variable, and significantly negatively correlated with DBP and positively with MV when DBP was adopted as an explanatory variable.

MT-ATI significantly negatively correlated with gender (greater in women) and CRAE and positively with age and DBP, while MV-ATI was significantly negatively correlated with gender (greater in women) and positively correlated with DBP when DBP was adopted as an explanatory variable.

## Discussion

In the current study, we found some of the LSFG parameters to be significantly correlated with the quantitative indices of ocular circulation, that is, MT, MV, CRAE, or the ONH structural parameters, such as disc and β-PPA area, after adjustment for systemic parameters such as age or blood pressure.

### Skew

**Figure 1:**
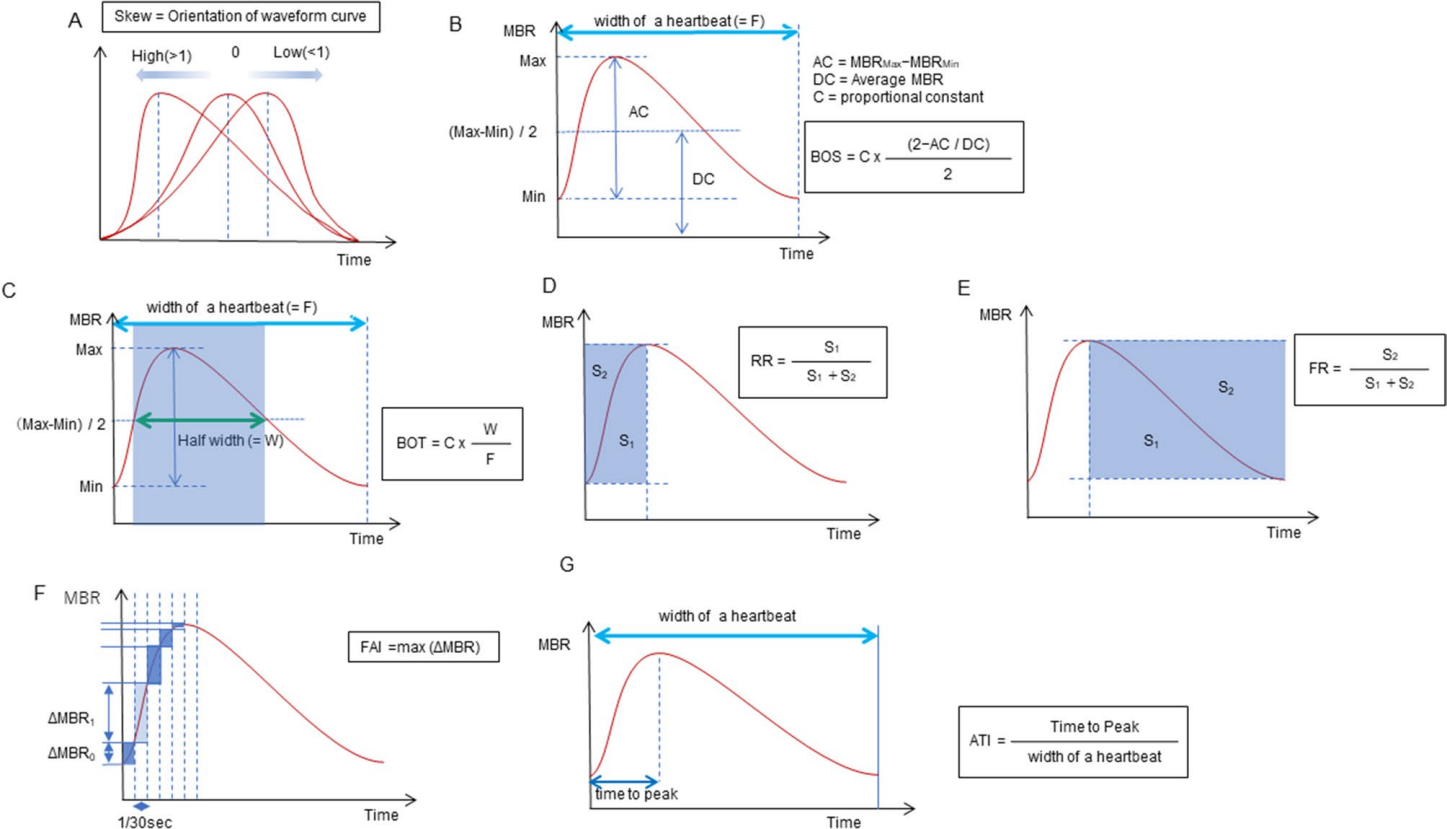
The pulse waveform analysis of the seven parameters. Skew represents the asymmetry of the waveform. A skew value of 0 describes a perfectly symmetrical waveform shape. If the peak comes faster and the distribution is leftward, the skew increases, and if the peak is slower and the distribution is rightward, the skew decreases (**A**). The blowout score (BOS) is considered an index of the blood flow that is maintained in one heartbeat (width of a heartbeat) and is calculated using the difference of the maximum and the minimum MBR as well as the average MBR. A high BOS indicates a high constancy of blood flow during the cardiac cycle (**B**). The blowout time (BOT) represents the ratio of the half-width in one heartbeat (width of a heartbeat). High BOT is an indicator of well-maintained perfusion during the cardiac cycle (**C**). The rising rate (RR) is the proportion of the area of S1 to S1 + S2 (**D**). The falling rate (FR) is the proportion of the area of S2 to S1 + S2 (**E**). The RR and FR characterize the steepness of the ascending and descending portion of the waveform curve, respectively. Higher values indicate a more sudden increase or decrease of MBR. The flow acceleration index (FAI) represents the maximum change in the increasing MBR in 1/30 s (**F**). The acceleration time index (ATI) is the ratio of the duration of the time to reach a peak (width to reach peak) in one heartbeat (width of a heartbeat) (**G**).

Skew quantifies the asymmetry of the waveform distribution. If the distribution of the waveform is leftward, Skew is higher (Fig. 1A). The value of Skew also increases as the slope of the waveform after the peak becomes steeper, indicating a quicker drop-off in the blood flow after the peak. Both MT- and MV-Skew showed a significant positive correlation with age and a negative correlation with DBP and HR (Table 4). Since the buffering capacity of the large arteries (i.e., the Windkessel effect) diminishes with increasing age, because of arteriosclerosis and reduction of elastic fiber, peripheral ejection of arterial blood occurs mostly during systole and is decreased during diastole^33,34^, resulting in higher Skew values, being compatible with the previous results obtained for age and HR^16,21^. An inverse correlation of the MT- or MV-Skew to age and DBP warrants discussion. In general, there was a rise in SBP and DBP with age. However, after the age of 50–60 years, DBP declined, yielding a rise in the pulse pressure (SBP–DBP)^35^. It was considered that the initial increase in SBP and DBP was due to an increase in peripheral vascular resistance, and a decrease in DBP observed after the age of 50 years was due to the increase in aortic stiffness^35^. Therefore, it seems reasonable that the opposite direction of correlation of MT- or MV-Skew to age and DBP was seen under certain conditions. Higher MV-Skew was showed to be associated with lower HR in the current study, being compatible with the water-drinking test (WDT) result^24^, because MV-Skew reportedly increased 40 min after the WDT^24^, which probably reflected a decrease in HR associated with the WDT^36^. Regarding the correlation to ocular factors, we found that the MT-Skew was significantly negatively correlated with the β-PPA area (Table 4). In previous reports, MT-Skew was significantly lower in patients with normal-tension glaucoma (NTG)^24^ and primary open-angle glaucoma (POAG)^26^ than in those with normal eyes. It is well known that a greater β-PPA area is a risk for progression of glaucoma^37,38^, being associated with the extent of glaucomatous visual field damage^39–41^. Therefore, lower MT-Skew associated with a greater β-PPA area seems compatible with an increase in glaucomatous damage or risk for glaucoma, and also with the previous results of lower MT-Skew reported in NTG^24^ and POAG^26^. It is possible that after adjustment for age, DBP, and HR, MT-Skew could reflect circulatory changes in the ONH tissue associated with the β-PPA area. MT is an indicator of blood flow in the ONH peripheral circulation supplied by the short posterior ciliary artery^10–12^ and MV is an indicator of blood flow in the major retinal vessels supplied by central retinal artery^13–15^. Thus, the difference in the correlation of β-PPA area between MT and MV-Skew is thought to be, at least in part, associated with the difference in the supplying arterial system.

### Blowout score

The BOS indicates the variation in the MBR during the systolic and diastolic periods, and a higher BOS indicates a higher constancy of blood flow during the cardiac cycle (Fig. 1B). In the current study, MT- and MV-BOS showed a significant negative correlation with age and a positive correlation with DBP and HR in healthy participants (Table 4). These results suggest that the decrease in MT- or MV-BOS systemically reflects an age-related increase in the stiffness of large arteries, consistent with the previous results^6,17^, and are compatible with a very strong negative correlation of MT- or MV-BOS to the resistivity index (Spearman’s correlation coefficient: − 0.997– − 0.991). The opposite direction of correlation of MT- or MV-BOS to age and DBP may be explained as previously described for MT- or MV-Skew. Thus, MT- and MV-BOS are considered to be useful waveform parameters to obtain information on the systemic circulatory status.

In addition, we newly found that MT-BOS was also significantly negatively correlated with CRAE (Table 4). Since many studies have shown that a narrower CRAE is associated with aging and cardiovascular diseases^42^ and also with glaucoma and its development^43–46^, the current finding implied that a higher MT-BOS may be suggestive of compromised retinal circulation. Gardiner et al.^26^ reported that a lower MT-BOS in glaucoma suspect/fellow eyes without a functional loss than in normal eyes was associated with a higher MT or higher blood flow in these eyes than in normal eyes. Conversely, Takeshima et al.^27^ reported that MT-BOS increased significantly after trabeculectomy, which could suggest an increased ONH circulation resulting from the ONH vascular bed reaction (in which the autoregulation mechanism was compromised^47,48^) to increased perfusion pressure due to IOP reduction. A strong negative correlation of MT-BOS to the resistivity index also supports the hypothesis of Takeshima et al. Thus, it seems currently difficult to use a lower MT-BOS value as an indicator of compromised ONH circulation.

### Blowout time

The BOT represents the length of time that the wave maintained more than half of the mean of the maximum and minimum MBR during a heartbeat (Fig. 1C). In the current study, MT- and MV-BOT showed a significant negative correlation with age, and MT-BOT showed a positive correlation with HR (Table 4). Shiba et al. suggested that the decrease in MT-BOT reflected the age-related increase in the stiffness in large arteries and/or systemic vascular resistance^21^, being compatible with its negative correlation with age in the current and previous results^6,16,17,21^. Since no significant correlation to ocular parameters was currently found for MT- and MV-BOT, these waveform parameters may be more sensitively reflect systematic vascular changes associated with aging rather than ocular circulatory status.

### Rising rate and falling rate

The RR is defined as the ratio of the waveform area before the peak (S1) to the entire area (S1 + S2) before the peak (Fig. 1D). The FR is defined as the ratio of the waveform area after the peak (S2) to the entire area after the peak (S1 + S2) (Fig. 1E). Higher values indicate a more sudden increase or decrease in the MBR. Currently, we found that both MT- and MV-RR were significantly negatively correlated with HR, and MV-RR to gender (higher in women) and smoking history (Table 4). The HR is strongly associated with LSFG pulse waveforms because the frame number (the total number of frames was 118 frames/4 s in the present study) reflects the duration of a heartbeat. Accordingly, the higher the HR, the shorter the duration of one heartbeat and the lower the number of frames per heartbeat. Thus, changes in the MT- or MV-RR will be correlated with changes in HR. Previous studies have reported that women have higher MT-^6^ and MV-RR^17^ compared to men^7–9^, which was consistent with the current result.

In the current study, both MT- and MV-FR showed a significant positive correlation with age, and MT-FR was negatively correlated with HR (Table 4), consistent with the previous results obtained for correlations between both MT-^6,16,17^ and MV-FR^17^ and age. An increase in MT- or MV-FR, indicating a more sudden drop-off in the blood flow after the peak, may reflect age-related stiffness in the large arteries, as in the cases of Skew and the BOS. The reported increase in MT-FR 10 min after WDT in healthy participants may be explained by a significant negative correlation of MT-FR with HR, since it decreased after WDT^36,49^. The current study could not find a significant correlation between MT-, MV-RR, and FR and any of the ocular parameters, which suggested limited usefulness of these parameters in studying ocular circulatory status.

### Flow acceleration index

The FAI describes the maximum change among all frames in a rising curve (Fig. 1F). The correlation of MT- or MV-FAI with systemic parameters has not been reported yet. We found that both MT- and MV-FAI showed significantly negative correlations with SBP and DBP and positive correlations with MT and MV, respectively, and with CRAE. Further, MT-FAI showed a significantly negative correlation with disc area, and MV-FAI was negatively correlated with age and HR (Table 4). As discussed above, a negative correlation of MT- or MV-FAI with SBP and DBP is considered to represent the effects of an age-related increase in the stiffness of large arteries on the waveform parameters. Many previous studies have reported a lower MT in NTG eyes^25,50,51^, and MT-FAI was also reported to be significantly lower in NTG eyes than in normal eyes^25^. Both MT and MT-FAI were reported to be significantly higher in glaucoma suspect/fellow eyes without a functional loss than in normal eyes^26^. These results are compatible with the significant positive correlation between MT-FAI and MT found in the current study. A previously reported negative correlation between CRAE and SBP or DBP^42^ also seems consistent with the significant negative correlation of MT- or MV-FAI with SBP and DBP, and the positive correlation of MT- or MV FAI with CRAE observed in the current study. Taken together, MT- and MV-FAI could be ophthalmologically useful LSFG pulse waveform parameters to further characterize the ONH tissue circulation complementing MT, a quantitative index of the ONH tissue blood flow^10–12^, and MV, the blood flow through major retinal vessels^13–15^, respectively. That is, higher values of MT- and MV-FAI indicate advantageous conditions in the ONH tissue circulation and retinal blood flow, respectively. A significant negative correlation between MT-FAI and disc area may be difficult to explain. Histological studies have demonstrated that optic nerve fiber count significantly increased with the enlargement of the optic disc size, but the nerve fiber density per disc area decreased when the disc area increased^52,53^. If local circulation is associated with the density of nerve fibers, it may be possible that the ONH tissue circulation, that is, the MBR measurement results from a unit area (one pixel) of the ONH or MT, and consequently its waveform parameter, MT-FAI, may be affected by the disc size. Whatever the causes for the correlation between MT-FAI and disc area, this result suggests that MT-FAI needs correction for disc area for inter-individual comparison. A significant negative correlation between MV-FAI and HR may be explained by the partial dependency of the FAI on the number of frames per heartbeat, as in the case of MT- or MV-RR.

### Acceleration time index

The ATI is derived from the duration of time taken before reaching the peak, and a higher MT-ATI represents a delay in the peak of the waveform (Fig. 1G). MT-ATI showed a significantly positive correlation with age and a negative correlation with CRAE, and both MT- and MV-ATI showed a significantly positive correlation with DBP and a negative correlation with gender (higher in women) (Table 4). A positive correlation of MT-ATI with age and DBP would be compatible with an age-related increase in the stiffness in large arteries, suggesting that a higher MT-ATI is associated with an unfavorable status of the systemic circulation. Conversely, MV-ATI reportedly showed a negative correlation to left ventricular mass^32^ and a higher MT- or MV-ATI in women was reported to be associated with lower left ventricular mass^11^. Since increased left ventricular mass, indicating increased left ventricular hypertrophy, was associated with increased risk of cardiovascular disease morbidity and mortality^54,55^, a higher MT-^6,17^ or MV-ATI^17^ in women currently and previously found suggests that higher MT- or MV-ATI favored left ventricular function, which is not compatible with a positive correlation of MT- and MV-ATI to age currently and previously found^6,11^. MT and MV have been reported to be higher in women than in men^7–9^. Thus, a higher MT- or MV-ATI in women may suggest that a higher MT- or MV-ATI favors the ONH tissue circulation and blood flow through major retinal vessels, respectively. On the other hand, a higher MT-ATI was reported in NTG eyes^24^, and MT was generally lower in NTG eyes^25,50,51^. A higher MT-ATI was currently found to be significantly associated with lower CRAE. Taken together, these results suggest that a higher MT-ATI may be associated with a compromised ONH or retinal circulation. Thus, as far as the current and previous results are concerned, it seems difficult to determine how MT- or MV-ATI reflects systemic or ophthalmic circulatory status, and further studies are needed to characterize MT- or MV-ATI as an indicator of systemic or ophthalmic circulatory status.

Our study had several limitations. First, we used disc parameters that were evaluated by planimetric methods. The current photographically determined β-PPA area included the γ-zone PPA and disc area could be better evaluated using spectral-domain optical coherence tomography (OCT)^56,57^. However, until now, the effects of β-PPA area on glaucoma have been investigated using photographs in many studies. Moreover, it is not common to measure the β-PPA area using OCT in routine clinical practice, but rather to evaluate the β-PPA area using photographs or ophthalmoscopy. Therefore, we believe that the current findings obtained for photographically determined β-PPA have clinical and practical significance. Second, the ellipsoidal bands needed to be fitted to determine the ONH margins in our participants. Thus, participants for whom the contours deviated from the ellipsoid, such as those with highly myopic discs, were not included in this study. Therefore, these results may not be relevant especially for individuals with high myopia, which is relatively common in Japan. Finally, the average age of the participants in the current study was relatively young. Therefore, the influence of age or blood pressure might not have been sensitively evaluated in the current study.

In summary, caution is needed to adopt some of the LSFG pulse waveform parameters, such as the BOS and ATI, in studying ocular circulation, since the results reported so far, including those from the current study, have yielded conflicting correlations between these waveform parameters and the ocular circulatory status. The BOS, BOT, RR, and FR may be used to obtain information on the systemic circulatory status, as a correlation to a quantitative index of ocular circulation such as MT, MV, or CRAE could not be detected as far as the current study was concerned. Conversely, MT-Skew was found to significantly correlate with β-PPA area, which was closely related to glaucoma damage^24,26,37,38^ and the FAI were found to significantly correlate with the quantitative indices of ocular circulation after adjustment for other confounding factors, which was compatible with the correlation of this parameters to the systemic circulatory status. Therefore, Skew and FAI were considered to have the potential to yield additional information which has ophthalmological implication.

## Methods

### Participants

This was a prospective cross-sectional study conducted at multiple facilities. The participating research facilities in Japan were the Fukui-ken Saiseikai Hospital (Fukui), Kanazawa University Hospital (Kanazawa), Kitazato University Hospital (Kanagawa), Tajimi Iwase Eye Clinic (Gifu), Toho University Ohashi Medical Center (Tokyo), Toho University Omori Medical Center (Tokyo), and Tohoku University Hospital (Sendai). This study was approved by the ethics committee of Toho University Medical Center Ohashi Hospital (No.15–86), a representative facility, and was also approved by the institutional review boards of each facility. All study conduct adhered to the tenets of the Declaration of Helsinki. Written informed consent was obtained from all participants.

Self-reportedly healthy participants, between 30 and 80 years of age, underwent a comprehensive screening examination, including a slit-lamp examination, indirect dilated fundoscopy, and measurement of IOP using a Goldmann applanation tonometer. The exclusion criteria were as follows: best-corrected visual acuity ≤ 20/40; spherical refractive errors >  ± 6.0 diopters (D); refractive cylindrical errors > 2.0 D; axial length > 26.5 mm; IOP > 21 mmHg; narrow peripheral anterior chamber with a Van Herick grade of ≤ 2; significant opacities of the optical media (e.g., corneal scars, clinically significant cataract according to the lens opacities classification system (LOCS) III criteria^58^); an abnormal visual field test result according to the Anderson-Patella criteria^59^ or an unreliable visual field test result (false positives or false negatives > 20%, or fixation loss > 30%); a history of intraocular eye diseases and intraocular surgery; a history of diabetes mellitus or cardiovascular disease; SBP > 150 mmHg and/or DBP > 90 mmHg; and intake of oral medications that may affect ocular circulation (calcium antagonists, α-1 or β blockers, or sildenafil).

### Measurement protocol

The LSFG measurement protocol was as follows. (1) Participants were interviewed to record medical history, including oral medication, and smoking history to ensure that they did not meet the exclusion criteria. (2) On measurement days, smoking was prohibited, and participants were instructed to abstain from caffeine-containing beverages. (3) Height and weight were measured. (4) Ocular examinations including measurements of refraction, best-corrected visual acuity, corneal curvature, axial length (AL), IOP, standard automated perimetry, OCT, and color fundus photography were conducted. (5) The pupils were dilated by topical instillation of 0.4% tropicamide 30 min before the LSFG examination. Measurements were obtained in the afternoon, and examination within 2 h after a meal was avoided. (6) BP measurement was performed after a 10 min resting period. After a further 10 min resting period in a dark room, three consecutive LSFG measurements were performed. During the measurement period, participants were encouraged to keep their breath steady. Artificial tear drops were instilled if the tear film was unstable because of dryness of the eye.

### Measurements of pulse waveform parameters in laser speckle flowgraphy

**Figure 2:**
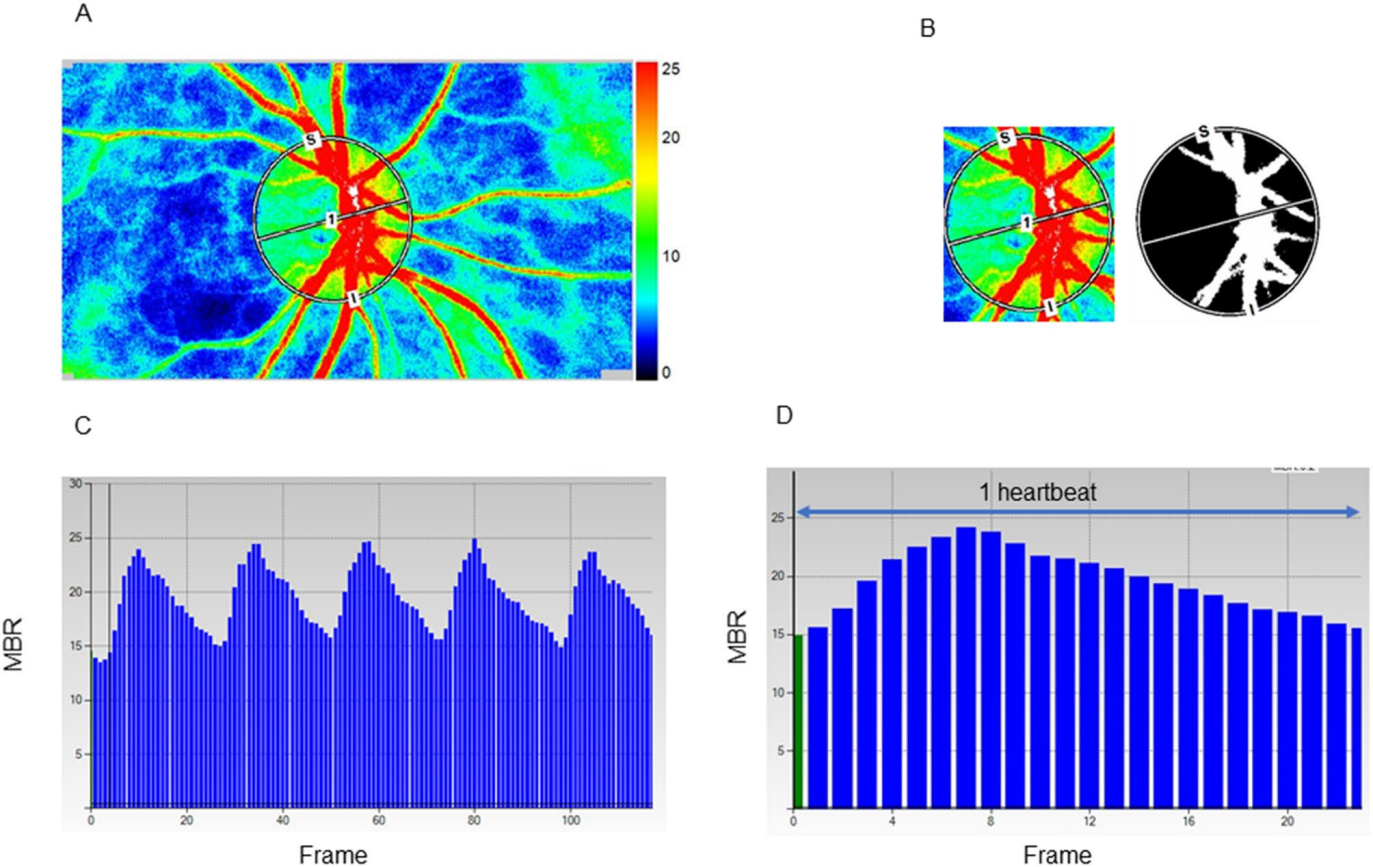
Analysis of pulse waveforms for the optic nerve head (ONH) using laser speckle flowgraphy (LSFG). Representative color-coded composite map (**A**). The mean blur rate (MBR) was automatically calculated with the help of the circle rubber band at the ONH. A binary format image for segmentation between the tissue area (black area) and the vessel area (white area) on the ONH (**B**). Pulse waves showing changes in the MBR, which is tuned to the cardiac cycle for 4 s. The total number of frames is 118 in a scan (**C**). Normalization of the change in the MBR in one heartbeat. This shape of MBR is a pulse waveform (**D**).

ONH blood flow was evaluated using LSFG (LSFG-NAVI; Softcare Ltd., Kyushu, Japan), and the parameters were calculated by LSFG Analyzer software (ver. 3.2.3.0, Softcare Co.). The principle and methods of LSFG have been described in previous studies^5^. Briefly, the instrument comprises a fundus camera equipped with a diode laser (wavelength, 830 nm) as the light source and a digital charge-coupled device camera (resolution, 750 × 360 pixels). LSFG automatically detects errors due to blinking and fixation. And we have further deleted data for which measurement results were not available due to heart rate analysis errors. The ONH margin was manually drawn with an ellipsoidal band (Fig. 2A), and the position of the ONH was saved on the system. The accompanying LSFG software automatically divided the ONH area into the large visible vessels and capillary (tissue) area using a binarization (cross-section analysis) (Fig. 2B) and provided the values for the ONH tissue-area MBR (MT), ONH vessel-area MBR (MV), and all-area MBR (MA). The primary output parameter of LSFG, the MBR, represents the relative blood flow velocity and is expressed in arbitrary units (AUs)^5^. After collecting the LSFG data from each facility, the ONH margins for all participants were determined with the ellipsoidal bands by a single experienced operator (T.S.) while referring to the fundus photograph. The time changes in MT or MV with the cardiac cycle were also recorded automatically for 4 s with a total number of frames of 118 (Fig. 2C) and images corresponding to identical phases within the duration of one heartbeat were synthesized to one image sequence (Fig. 2D). By delineating an MT or MV waveform by plotting the MTs or MVs derived from each frame (Fig. 2D), several shape parameters of the MT or MV pulse waveform, which are synchronized with the cardiac cycle^7,16^, were output by LSFG. We focused on seven MT and MV pulse waveform parameters in the current study: Skew (Fig. 1A), BOS (Fig. 1B), BOT (Fig. 1C), RR (Fig. 1D), FR (Fig. 1E), FAI (Fig. 1F), and ATI (Fig. 1G) in MT and MV. We adopted an average of three outputs of the MT and MV waveform parameters in the analysis. The resistivity index and fluctuation were considered to be represented by the BOS because of their high inter-correlations with the BOS (Spearman’s correlation coefficient: − 0.997– − 0.967).

### Measurement of clinical parameters

Standard automated perimetry was performed with the Humphrey visual field analyzer 24–2 Swedish interactive threshold algorithm-standard strategy test (Carl Zeiss Meditec, Dublin, USA). The AL was measured with an optical biometer (IOLMaster; Carl Zeiss Meditec) or OA-2000 (Tomey, Nagoya, Japan). The data from OA-2000 were transformed to values yielded by the IOLMaster according to previous studies^60,61^. Color fundus photographs were acquired with TRC-50DX (Topcon, Tokyo, Japan) or TRC-NW7SF (Topcon). OCT measurements were performed with one of the following instruments based on the availability in each facility: RS-3000 (NIDEK, Tokyo, Japan), 3D OCT-2000 (Topcon), DRI OCT Triton (Topcon), and RTVue-XR Avanti (Optovue Inc., Fremont, CA, USA). Only the cup/disc area ratio values were used for the analysis of the OCT measurements.

### Disc and β-peripapillary atrophy (PPA) area measurements

The details of the planimetric method used in this study have been reported previously^62–64^. An experienced ophthalmologist (A.I.) examined all color fundus photographs. After correcting for magnification based on a modification of Littman’s method provided by the manufacturer (Topcon), planimetric parameters, disc, and β-PPA areas were calculated using image analysis software (JGSTKDiscAnalysisSoft; Topcon). In the current study, the OCT measurements were performed using one of the following instruments: RS-3000 (NIDEK), 3D OCT-2000 (Topcon), DRI OCT Triton (Topcon), or RTVue-XR Avanti (Optovue Inc.). Different OCT instruments might employ different algorithms to determine the clinical optic disc margin and to correct magnification of the fundus image; thus, we used the disc area of each eye determined using a fundus photograph and used only the cup/disc area ratio yielded with each OCT instrument to calculate the cup and rim areas for each participant’s eye using the following formula: cup area = disc area provided by the fundus photograph × cup-to-disc area ratio provided by each OCT instrument; rim area = disc area − cup area of the same eye.

### CRAE and CRVE measurements

CRAE and CRVE were determined using the photographs obtained with the Topcon fundus cameras, according to a previously reported method^65^.

### Statistical analyses

All data are shown as the mean ± standard deviation. The normality of the data was examined using the Kolmogolov-Smirnov test. We evaluated the contributing factors for the seven LSFG pulse waveforms: Skew, BOS, BOT, RR, FR, FAI, and ATI in MT and MV using univariate and multivariate linear mixed-effect modeled regression analyses to adjust for the confounding effects of other factors and the correlation between two eyes of a subject. Independent variables were age, gender, smoking history, body mass index, SBP, DBP, HR, IOP, AL, disc area, rim area, β-PPA area, MT for MT-waveforms, MV for MV-waveforms, CRAE, and CRVE. The factors that showed *P* values less than 0.2 in the univariate analyses were included as independent variables in the multivariate linear mixed-effect modeled regression analysis. If SBP and DBP simultaneously yielded *P* values < 0.2 in the univariate analyses, they were included separately in the multivariate analyses. Multivariate analyses included 9 or less independent variables. It was confirmed that no independent variables included in the multivariate analysis showed correlation coefficients > 0.7. Since 10 samples or more are required for one dependent variable in multivariate analysis^66^, the sample size of 111 eyes is considered to be within the appropriate range. All analyses were performed using the statistical software SPSS version 24.0 for Windows (IBM Corp., Armonk, NY). Statistical significance was considered at *P* < 0.05.

## Data Availability

The datasets generated during and/or analyzed during the current study are available from the corresponding author on reasonable request.
